# A Gender-Affirming Approach to Contraceptive Care for Transgender and Gender-Diverse Patients

**DOI:** 10.1097/og9.0000000000000003

**Published:** 2024-03-26

**Authors:** Dustin Costescu, Carys Massarella, William J. Powers, Sukhbir S. Singh

**Affiliations:** McMaster University, Hamilton, and Ottawa Hospital Research Institute, Ottawa, Ontario, Canada; and Powers Family Medicine, Farmington Hills, Michigan.

## Abstract

Transgender and gender-diverse (TGD) reproductive health care is a field with an abundance of misinformation and a paucity of quality literature available for both health care professionals and their patients. Clinicians often receive limited education and training in this area, and TGD individuals face many barriers to reproductive health care, including the lack of gender-affirming, inclusive, and knowledgeable clinicians as well as concerns about gender biases. As such, TGD individuals often feel uncomfortable discussing their reproductive health with their health care professionals and are deterred from seeking the appropriate care they need. Contraceptive counseling is a key component of reproductive health care but is often neglected in discussions between TGD patients and their health care professionals. Clinicians must strive to meet the contraceptive health care needs and desires of TGD patients in a gender-affirming manner within a safe and accepting space. Here, we summarize the current reproductive health care landscape and provide contemporary perspectives on how to improve contraceptive care for TGD individuals.

## Abstract 2

Health care professionals should ensure that the contraceptive needs of their transgender and gender-diverse patients are met in a gender-affirming manner.

The transgender and gender-diverse (TGD) umbrella encompasses a wide range of gender identities, including (but not limited to) transgender men, transgender women, gender nonconforming, and gender nonbinary. A transgender person is someone whose gender identity differs from the gender they were assigned at birth. Recently, there has been a shift in cultural, political, and social perspectives toward a broader acceptance of gender as a continuum.^1^ This view suggests that a person's gender may not be aligned with the sex they were assigned at birth and can fall anywhere (or everywhere or nowhere) along the gender spectrum.^2^ Table 1 shows a comprehensive, but not exhaustive, list of terms related to the TGD population.

**Table 1.** Glossary of Terms Related to Transgender Health Care*

Term	Definition
Assigned female at birth, AFAB^1^	A person, irrespective of gender identity, whose sex assignment at birth resulted in a declaration of “female”
Agender^42^	A person who identifies as not having a gender or being outside of the gender continuum; a subcategory of gender nonconforming
Assigned male at birth, AMAB^1^	A person, irrespective of gender identity, whose sex assignment at birth resulted in a declaration of “male”
Cisgender, cis^42,43^	A person whose gender identity corresponds with that of their sex assigned at birth
Cisnormativity^44^	The assumption (often unconscious) that all individuals are cisgender
Female to male, FtM^43,44^	A person whose gender identity is male but whose sex assigned at birth was female; the term transgender man is often preferred because this places the emphasis on the individual's current gender identity
Gender-affirming^1,42^	Behaviors or interventions that affirm a person's gender identity
Gender binary^1,42,44^	The classification of gender into two distinct, opposite categories: male and female
Gender dysphoria^1,42,44^	A sense of psychological discomfort or distress that a person may feel as a result of a disparity between their gender identity and their sex assigned at birth
Gender expression^1,42–44^	The way in which a person expresses their gender identity, normally through a combination of appearance and behavioral factors
Gender fluid^42^	A person whose gender identity, gender expression, or both fluctuate over time; a subcategory of gender nonconforming
Gender identity^1,42–44^	The personal sense of one's own gender
Gender neutral^42–44^	Not referring to male or female genders; gender-neutral language is often more inclusive, particularly for transgender patients
Gender nonconforming, genderqueer, gender-diverse^42–44^	A person whose gender identity does not subscribe to conventional gender distinctions but identifies outside the gender continuum, between the binary gender categories of male and female, or as a combination of male and female genders
Heteronormativity	The assumption (often unconscious) that all individuals are heterosexual
Male to female, MtF^43,44^	A person whose gender identity is female but whose sex assigned at birth was male; the term transgender woman is often preferred because this places the emphasis on the individual's current gender identity
Minority stress^42^	The additional stress experienced by members of stigmatized minority groups due to the prejudice and discrimination that they face
Nonbinary^42,44^	A person whose gender identity sits outside of the gender binary; a subcategory of gender nonconforming
Preferred gender honorific	The honorific or form of address that a person uses and wishes others to use; this may include gendered terms (eg, Mr, Mrs, Miss, Ms) or gender-neutral terms (eg, Mx)
Preferred gender pronouns^44^	The set of pronouns that a person uses and wishes others to use to reflect their gender identity; these may include gender-neutral pronouns (such as they or them) or neopronouns (such as ze or zir)
Transfeminine^42^	A person who was assigned male at birth but whose gender identity is on the feminine spectrum
Transgender, trans^1,42–44^	A person whose gender identity does not correspond with that of their sex assigned at birth
Transgender and gender-diverse, TGD^1,42–44^	A person whose gender identity does not correspond with that of their sex assigned at birth, including those with gender identities that lie outside the gender continuum, between the binary gender categories of male and female, or as a combination of male and female genders
Transgender woman, trans woman^1,42–44^	A person whose gender identity is female but whose sex assigned at birth was male
Transgender man, trans man^1,42–44^	A person whose gender identity is male but whose sex assigned at birth was female
Transmasculine^42^	A person who was assigned female at birth but whose gender identity is on the masculine spectrum
Transphobia^1,42–44^	Dislike, fear, discrimination, or prejudice against transgender people
Transsexual^1,42–44^	A historic term for transgender people, usually used to denote those who have gone through gender-affirming therapy; most transgender people prefer the term transgender, so health care professionals should avoid the term transsexual unless this is stated as the patient's preference
Queer^42,44^	An umbrella term for people who are not heterosexual or are not cisgender; although once used pejoratively, this word has now been reclaimed and is used to describe a broad spectrum of nonnormative sexual or gender identities

* Please note that this list is not exhaustive; there are many terms used to describe and express gender identity.

The TGD population represents a minority group, and the exact proportion of TGD people in the general population remains unknown. One study estimated it to be approximately 355 per 100,000 people,^3^ and another proposed a range between 0.4% and 1.3% of the global population.^4^ Regardless, this community experiences significant minority stress, that is, stress resulting from discrimination and stigma, and research has reported a significantly higher prevalence of anxiety and depression among the TGD population than the cisgender population (see definition in Table 1).^5,6^ Marginalized and minority groups historically have struggled to meet their health care needs, but recent efforts have been heightened to be more inclusive of the TGD community in health care settings. However, there remains a paucity of research and clinical guidelines specifically addressing health care for TGD individuals, particularly with regard to family planning and contraception.^7^

## BARRIERS TO REPRODUCTIVE HEALTH CARE FOR THE TGD POPULATION

Despite increasing support, visibility, and acceptance, the TGD community still faces significant barriers in their ability to access quality reproductive health care.^8–10^ For example, TGD people assigned female at birth (AFAB; including, but not limited to, transgender men) can become pregnant and, therefore, may require contraception, yet they often face obstacles in accessing satisfactory contraceptive care.^8,11–13^ Recent surveys of AFAB TGD patients have confirmed that few patients use highly effective contraceptive methods (such as intrauterine devices [IUDs] or implants), and many use no contraception at all.^14^ Barriers to contraceptive care for TGD individuals include a lack of insurance coverage; a lack of gender-affirming, inclusive clinicians; and concerns that their health care professional may hold cis- and hetero-normative assumptions or gender identity–related biases (whether intentional or not).^8,11–13^ Regardless of where a patient identifies on the gender spectrum, everyone should have equitable reproductive health care and access to health care professionals who can tailor their contraceptive provision to individual needs.^7^

Clinicians typically are provided with insufficient education, training, and guidance regarding sexual and reproductive health care for TGD individuals, particularly in contraception and pregnancy prevention.^8,11–13,15^ Coverage of health care topics relevant to TGD individuals in the literature is scarce, and further studies are needed.^8,11,15,16^ As a result, there are very limited expert recommendations to support the education of health care professionals who are less experienced in treating TGD patients. This translates into another barrier to health care for the TGD population, because many clinicians lack confidence in providing adequate care for their TGD patients.^10,15^ Consequently, TGD people are less likely to seek medical help than the cisgender population,^9^ and those who do visit clinicians regarding their reproductive health often are frustrated by their lack of experience and understanding.^9,10^

## TACKLING THE CHALLENGES IN PROVIDING QUALITY REPRODUCTIVE HEALTH CARE FOR THE TGD POPULATION

Although education and resources relating to the clinical care of TGD people are limited, clinicians are driven to provide quality care for their patients and, therefore, have a high interest in seeking out this information.^8^ The U.K. Faculty of Sexual and Reproductive Health care released a statement in 2017 that provided guidance on contraceptive choices for TGD and nonbinary people.^17^ In light of the paucity of evidence in the existing literature, this statement was based largely on the consensus of experts in this field and offered insights into the efficacy and safety of contraceptives among TGD individuals. Similarly, the Society of Family Planning has developed recommendations by integrating the unique needs of TGD patients with what is known and established in cisgender patients.^18^ Other guidelines for the care of TGD people often focus on fertility rather than pregnancy prevention.^1,11^ In 2019, the World Health Organization released an update to the International Statistical Classification of Diseases and Related Health Problems, stating that it is crucial for TGD individuals to have equal access to health services.^19^

As the TGD community continues to receive greater visibility and support, clinicians may begin to see TGD patients in their clinical practices more frequently. Thus, improving access to and provision of contraceptive care for TGD individuals has become ever more important. Here, we discuss the essential aspects of contraceptive care for the TGD community, including pregnancy prevention and menstrual regulation, and provide recommendations for how health care professionals can tailor care to their TGD patients.

## MISCONCEPTIONS ABOUT CONTRACEPTION AMONG TGD PEOPLE

Contraceptive counseling should be a routine component of reproductive health care but is often neglected in TGD patients, in part because health care professionals frequently are unaware that it is necessary.^10,13,15^ The prevalence of misinformation means that TGD patients often receive suboptimal, if any, contraceptive counseling, which limits their ability to ensure pregnancy prevention.^8,10,15^

Gender-affirming hormone treatments can have varying effects on fertility.^16^ In contrast to genital surgical procedures—including hysterectomy, oophorectomy, and orchiectomy—that are widely known to cause permanent loss of the ability to conceive, the effects of gender-affirming hormone therapy are mostly reversible. Contraception is an often overlooked area of health care in the TGD community, despite the fact that AFAB TGD individuals can still become pregnant and, therefore, require family planning and contraceptive counseling.^8,11–13,15,20^

Many misconceptions originate from the use of testosterone therapy, which is the most common type of gender-affirming therapy in AFAB TGD patients. Although testosterone therapy can reduce the probability of conception, the extent of this reduction and whether it is reversible after cessation of therapy remain unclear.^13,20^ Many clinicians and AFAB individuals are unaware of the fact that pregnancy is still possible even if the patient is amenorrheic when receiving testosterone therapy.^11,16,21^ Testosterone suppresses the hypothalamic–pituitary–gonadal axis and inhibits ovulation, particularly in new users, but data have suggested that this suppression can be overcome in long-term testosterone users and ovulation can occur.^22^ Some AFAB TGD individuals reported that they were misinformed by their health care professionals, who considered testosterone therapy as sufficient contraception.^11,13,21,23^ As such, there is a need for large-scale studies to systematically examine pregnancy rates (in person-months) among TGD patients undergoing testosterone therapy.^13,20^

Another common misconception regarding the use of testosterone therapy is the unwanted bleeding that AFAB TGD individuals may experience. Although guidelines state that testosterone therapy typically leads to amenorrhea,^1,18,24,25^ up to 25% of TGD patients using this therapy experience breakthrough bleeding.^26,27^ This can trigger gender dysphoria and be distressing for the patient. If clinicians are aware of this possibility, they can adequately counsel their patients, reduce their distress, and discuss alternatives for menstrual suppression if the patient desires.

It is also a common and false belief that hormonal contraceptives are unsuitable for AFAB TGD individuals, especially those using testosterone therapy.^1,11,21,28^ In fact, most hormonal contraceptives are safe and may be taken concurrently with testosterone therapy, thereby providing pregnancy prevention in addition to other benefits, such as menstrual suppression.^17^

Finally, although only AFAB patients are able to become pregnant, many health care professionals are unaware that many assigned male at birth (AMAB; including, but not limited to, transgender women) patients also require contraceptive counseling.^15^ Gender-affirming hormone therapy for AMAB TGD patients, which generally comprises estrogens and antiandrogens,^1,24^ can affect fertility by inhibiting spermatogenesis and reducing sperm motility and sperm count.^29^ The current literature is inconclusive regarding the long-term effects of these therapies on the fertility of AMAB TGD individuals. That being said, there is still the possibility of conception due to incomplete inhibition of spermatogenesis,^16^ and health care professionals should provide appropriate counseling so that AMAB patients can make an informed choice regarding contraception for themselves and their sexual partners.

## DETERMINING SUITABLE CONTRACEPTIVE METHODS FOR TGD PATIENTS

Each contraceptive method differs in a variety of ways, including duration of use, efficacy, bleeding profiles, method of use or insertion, and adverse event profiles. Contraceptive counseling is largely focused on the pretransition period, but continued counseling throughout and after the patient's transition process is also important. Health care professionals ideally would obtain the patient's sexual history to assess pregnancy risk and identify their needs and desires. These needs may differ from those of cisgender patients, and routine counseling procedures should be tailored accordingly.^7,8^ Contraceptive counseling should be patient-centered and holistic, taking into account individual characteristics, needs, and desires to select the most suitable contraceptive for the patient.

Contraceptive counseling should cover the patient’s personal preferences on factors such as duration and methods of use, as well as the extent of fertility preservation and menstrual suppression. Health care professionals also should discuss the advantages and disadvantages of different options in light of the individual's gender identity, needs, and potential dysphoria. Additionally, the effects of contraception on the patient’s gender-affirmation therapy, where applicable, should be considered.^7^ These factors, combined with relevant safety profiles, would provide the necessary information to assist patients in deciding on their ideal contraceptive method (Table 2).

**Table 2.** Advantages and Disadvantages of Methods for Contraception and Menstrual Suppression for Transgender and Gender-Diverse Individuals

Method	Advantages	Disadvantages
Combined hormonal methods (estrogen and progestin): •Pill •Vaginal ring •Transdermal patch	•Moderately effective (93% or higher)^30^ •Some evidence indicates this does not interfere with masculinization^45^,* •Continuous administration enables amenorrhea^46^	•May trigger gender dysphoria (estrogen) •Short-acting—requires regular use^30^ •Contraindicated with cardiovascular risk factors^30^ •Some evidence that this may counteract the masculinizing effects of testosterone^17^,*
Progesterone-only pill	•Moderately effective (90% or higher)^30^ •Safe to use in patients with medical comorbidities^30^	•Unscheduled bleeding and spotting^47^ •Short-acting—requires regular use^30^
Condom	•Nonhormonal^30^ •Protects against STIs^17^	•Less effective than hormonal methods (79% or higher)^30^
LNG-IUD	•Long-acting^30,36^ •Highly effective (greater than 99%)^30,36^ •May reduce menstrual cramps^30^ •May make periods lighter in the long term^30,36^ •Reduces number of menstrual bleeding days^30,36^ •5–20% of patients experience no bleeding after 1 y^30,36^	•Increased bleeding or spotting days in first 3–6 mo,^30,36^ which may contribute to gender dysphoria •Insertion process may trigger gender dysphoria
Copper IUD	•Long-acting^30^ •Highly effective (greater than 99%)^30^ •Nonhormonal^30^	•Heavier and longer periods in the 1st year of use^35,40,30^,† •Insertion process and bleeding profile may trigger gender dysphoria
Implant	•Long-acting^30^ •Highly effective (greater than 99%)^30^ •Safe to use in patients with medical comorbidities^30,33^ •Easily inserted and removed^30^	•Less well-tolerated due to the initially increased frequency and irregularity of bleeding vs LNG-IUD^30,36^
Medroxyprogesterone acetate injection	•Moderately effective (90% or higher)^30^ •Safe to use in patients with medical comorbidities^30^ •Suitable for patients looking for shorter-term contraception than the implant^40^	•Less well-tolerated and an increased frequency or volume of bleeding or both vs LNG-IUD^30^
Nonsteroidal aromatase inhibitor	•Suppresses menstrual bleeding^46^	•Menopause-like side effects^46^ •Negative effect on growth and bone health in adolescents^46^
Sterilization	•Highly effective (although it should be noted that vasectomy is safer and more effective than sterilization procedures for AFAB patients)^48,49^	•Permanent, not reversible^48,49^

STI, sexually transmitted infection; LNG-IUD, levonorgestrel-releasing intrauterine device; IUD, intrauterine device; AFAB; assigned female at birth.

* Please note that this evidence is conflicting.

† For those experiencing amenorrhea due to testosterone therapy, this increase in bleeding may not occur.

Transgender and gender-diverse patients undergoing gender-affirming surgery still require contraceptive counseling before their surgery. They may wish to use short-acting contraceptive methods, for example, the combined pill, the progestogen-only pill, vaginal rings, transdermal patches, or barrier methods such as condoms.^13,17^ Other TGD individuals may not wish to undergo surgery or may prefer medical interventions that preserve fertility. These patients may seek more benefits from long-acting reversible contraceptive methods, that is, hormonal and nonhormonal IUDs or implants.^13,17^ Thus, health care professionals should discuss the current and future wishes of patients with regard to reproductive health care. Patients should also be assured that they may cease or change contraceptive methods, or request additional counseling, at any point as their needs change over time.

Preferences around menstruation are another important factor to consider for AFAB patients; TGD individuals may wish to suppress menstruation for both practical and psychological reasons (eg, to prevent triggering gender dysphoria).^13^ The bleeding profiles of different contraceptives should be discussed to appropriately manage patient expectations and to help inform choices of contraceptive methods. In addition to bleeding, menstrual-like cramps and breast tenderness are not uncommon with hormonal contraception.^30^ These symptoms also may cause dysphoria and should be included when discussing possible adverse events. Effective and thorough counseling improves continuation and satisfaction with contraceptives^31,32^ and at the same time prevents or alleviates potential dysphoria.

As with cisgender patients, TGD patients may have underlying symptoms and conditions such as premenstrual syndrome or premenstrual dysphoric disorder, polycystic ovarian syndrome, endometriosis, or heavy menstrual bleeding. Hormonal contraception can provide relief for these conditions related to the menstrual cycle and should be factored into counseling conversations.^30^ Contraindications also should be included in discussions, using resources such as the World Health Organization’s medical eligibility criteria for guidance.^33^ For example, patients with a family history of breast cancer can use any method of contraception, whereas those with personal history of breast cancer should avoid hormonal contraceptives.^33^ Those with a history of deep vein thrombosis or pulmonary embolism are cautioned to avoid estrogen-containing contraceptives but may use progestogen-only methods.^33^

In addition to contraindications, consideration should be given to the preferences of AFAB patients when it comes to estrogen-based methods of contraception, such as the combined pill, especially because their use can trigger gender dysphoria in some patients.^1^ The extent to which estrogen-based contraceptives interfere with testosterone therapy–induced masculinization is still a topic of debate due to conflicting evidence.^1,17^ Transgender and gender-diverse patients may prefer non–estrogen-based methods, including the progestogen-only pill, hormonal levonorgestrel-releasing IUD (LNG-IUD), nonhormonal copper IUD, and medroxyprogesterone acetate contraceptive injection.

Of these options, the LNG-IUD is a popular choice for AFAB TGD individuals because of its suppressive effect on menstruation (Table 2).^1,13,14^ Evidence has shown that the LNG-IUD significantly reduced the number of bleeding days, and almost a quarter of users experienced amenorrhea after 3 years of use.^34–36^ However, this method was also associated with an initial increase in the number of bleeding and spotting days within the first 3–6 months,^34–36^ which may trigger gender dysphoria and increase dissatisfaction and discontinuation.^1,37,38^

Similarly, the nonhormonal copper IUD may contribute to gender dysphoria for AFAB TGD patients due to its bleeding profile.^1^ There were reports of significant increases in the number of bleeding days and volume in the first year of use,^35,39^ although some AFAB patients experiencing amenorrhea from testosterone therapy may not experience these increases in bleeding. Additionally, the copper IUD may be a more suitable option for patients who would prefer to avoid hormonal contraception.

Both hormonal and nonhormonal IUDs have high efficacy and a long duration of use.^40^ However, the placement procedure can be a concern because it may trigger dysphoria in TGD patients. Appropriate care can help minimize patient distress and dysphoria during the procedure. Box 1 provides our clinical tips for ensuring patient comfort and reducing dysphoria during IUD placement.^1^

Box 1. **Clinical Tips for Minimizing Transgender and Gender-Diverse Patients' Discomfort and Dysphoria During Intrauterine Device Placement**

- Before each step of the insertion procedure, calmly explain the actions that will be carried out.^50^
- Remind the patient that they can stop the procedure at any time if they wish.
- Use a small speculum when possible to minimize further discomfort and potential distress with the penetration of the instrument.^50^
- Perform other necessary examinations, such as Pap tests, at the same time as IUD insertion to minimize the number of gynecologic procedures.
- Assess the patient's vaginal capacity and uterine size, because they may be affected by hormonal therapies (which can also cause changes in vaginal epithelium).
- If ultrasound assistance is required for placement, consider patient preferences for evaluation. Transabdominal approach would be 1st-line (with a partially filled bladder). Endovaginal and endorectal ultrasonography, if required, should be performed with informed consent based on patient preference.^50^
- Anxiety and fear of pain increase the difficulty and pain of IUD insertion.^50,51^ Although most people experience no more than mild pain during insertion,^36,50,51^ pain management may be useful, particularly for patients who are anxious.
  ○ Local anesthetic (eg, lidocaine vaginal gel or an intracervical local anesthetic block with 2% lidocaine) has been shown to be beneficial in reducing pain with tenaculum and IUD insertion.^52,53^
  ○ Consider anxiety medication (eg, oral benzodiazepine) or pain medication (eg, NSAIDs) or both for patients with severe anxiety.
- Cervical dilation may be required if the IUD is difficult to insert.

As always, treat patients with empathy, respect, and dignity.

IUD, intrauterine device; NSAID, nonsteroidal anti-inflammatory drug.

When providing contraceptive counseling for TGD patients, it is essential for health care professionals to create a safe, inclusive, nonjudgmental, and gender-affirming clinical environment.^7,12^ This includes factors such as asking for and using the patient's chosen name and pronouns, avoiding assumptions, minimizing biases, and using gender-affirming language (Box 2).

Box 2. **Using Gender-Affirming Language: Examples From Contraceptive Counseling**

**Establishing Patient Rapport**

• “How would you like to be addressed?”
• “What would you like me to call you?”
• “What pronouns do you use?”

**Establishing Need for Contraception**

• “Is contraception something that you may need now or in the future, based on the types of relationships you have?”
• “Based on your relationships, is there a risk of pregnancy at this time?”
• “Is pregnancy something you would ever consider?”
• [For AFAB patients] “Pregnancy can still be a risk if you are taking testosterone, even if you don't get any bleeding.”
• [For AMAB patients] “While fertility decreases if you are taking estrogen, there is still a potential risk of getting a partner pregnant.”

**Discussing contraceptive options [For AFAB patients]**

• “Would you prefer to use a long-acting method (like IUDs or implants) or a short-acting method of contraception (like oral pills)?”
• “Does your current hormone therapy regimen prevent menstruation? If it does not, would you prefer a method of contraception that could prevent this?”
• “With IUDs or implants, your monthly bleeding pattern may change. This should settle after the first few months of use.”
• “Are you comfortable using a hormone-containing method of contraception?”
• “Would you prefer contraception that does not contain estrogen?”
• “In order to get an IUD, you will need a short procedure where it is placed internally in the uterus. Is this something you'd be comfortable with? Is there anything we can do to better accommodate you?”
• “If a contraceptive method isn't working for you and/or you decide you want to try a different method, you can come back any time to find a method that suits you better.”

AFAB, assigned female at birth; AMAB, assigned male at birth; IUD, intrauterine device.

Currently, data on contraceptive use in TGD patients are sparse. One study with 120 participants reported that only 20% of AFAB patients and 36% of AMAB patients were using contraception of any kind.^41^ Another study with almost 200 participants found that 60% were using contraception; of these, almost half were using condoms only and one-third were using oral pills.^11^ Few participants in either study were using longer-acting methods with higher efficacy; the reasons behind contraceptive choice were not explored in depth, although the dislike of estrogen-containing contraception and the concern of interfering with gender-affirming hormone therapy were cited as key reasons behind the discontinuation of combined pills. There is an unmet need to generate and share data on contraceptive use and patient preferences among the TGD population so that a stronger evidence base for effective counselling can be provided.

Additionally, it is important to bear in mind that contraceptive counseling can overlap with other fields of expertise, such as fertility-specific needs or psychiatric needs regarding gender dysphoria. Establishing a multidisciplinary team of clinicians with different areas of expertise that correspond to patients’ needs can permit the sharing of information and provision more holistic health care for TGD patients.^12^

## CONCLUSIONS

Current research and guidelines regarding contraceptive care for TGD people do not cover the full scope of the relevant issues for this population. Clinicians generally are not knowledgeable or comfortable in treating TGD patients due to a lack of training and guidance, and the language used in clinical practice often is not inclusive. This may deter TGD individuals from seeking the care they need. As health care professionals, we should strive to meet the contraceptive needs and desires of our TGD patients through effective counseling and respectful dialogue in a gender-affirming manner.
